
JOURNAL INFORMATION
==============================
NLM Title Abbreviation: Med Chir Rev
NLM Journal ID: 0034617

The Medico-Chirurgical Review

Publisher: Biomedical Journal Digitization Project

ARTICLE INFORMATION
==============================
PMCID: PMC4966185
PMID: 29917607
Article version: 1
Subjects: Articles

Hospital Practice

Print publication date: 1828 Jul 1
Volume: 9
Issue: 17
First page: 273
Last page: 288
1828 Jun 21
License: This work is free of known copyright restrictions. For more information, please see PMC Back Issue Digitization.
License URL: https://creativecommons.org/publicdomain/mark/1.0/

==============================

nothing.' Now this being a clear-headed man, I listened to what he said, and I did send him down some patients; *but he could not cure them*, though he certainly did relieve them."

When we found that only one case was to be selected, out of the innumerable cures of consumption through the medium of the stomach, we did expect that *it* would be what Goldsmith would have called a "*thumper*." But, after all, it turns out to be merely the transfer of some consumptive patients from Mr. Abernethy in London, to the "intellectual highlander," six miles in the country—where, by the way, the patients were "not cured," but only "relieved"—a very convenient phrase long used in hospitals, when patients go out *nearly* as well as when they came in! Whether the relief in question resulted from the stomach-medicines prescribed by the "intellectual highlander," or from the *change of air* from town to country, we leave to the judgment of those who have made any observations on the effects of such changes.

The lecturer admits that, in some cases, there may be disease both in the lungs and stomach—"but then, what does that prove? it does not prove which of the organs is the most diseased." Certainly not. Nor does it prove the priority of disease in one organ rather than in another. The following passage affords a fine specimen of what may be called the "*surgical pathology* of phthisis.

"But can consumption be cured? Odd bless me, that is a question which a man who had lived in a dissecting-room would laugh at: how many people do you examine who have lungs tubercular, but which are otherwise sound. What is consumption? It is tubercle of the lungs; then, if those tubercles were healed, and the lungs otherwise sound, the patients must get better. But if the enquirer shifts his ground, and says, it was the case I meant of tubercles over the whole of the lungs, why then he shifts his ground for no purpose; for there is no case which, when it has proceeded to a certain extent, can be cured. Therefore I say consumption may be cured, according to surgical principles, by creating local irritation on the side of the chest, and so on." 400.

Now the jet of the whole doctrine appears to be this:—stomach disorder is the primary state of disease in phthisis—ergo, cure the stomach-disorder, and you cure consumption. And as blue pill will cure stomach-disorder, why have recourse to any other medicine?—why trouble our heads about any other pathology, seeing that stomach-disorder is "the primary state of disease" of the lungs in pulmonary consumption? This reasoning is very plausible; and is very similar to that which was employed by the founder of our ancient and excellent ally the Turks. When Amru, the Mahomedan general, issued his mandate to burn the Alexandrian library, a deputation of literati waited on him, to dissuade him from his purpose. He asked them if the library contained any other books than the Koran; and being answered in the affirmative, he observed that, as the Koran contained every thing that was *necessary* to be known, so all books containing more than the Koran were *unnecessary*—and therefore he ordered the library to be burnt. The principles of Amru and Abernethy are precisely similar. Stomach-disorder is the cause of consumption—blue-pill and black-draught will cure stomach-disorder—ergo, all other pathology is erroneous—all other medicine is unnecessary.

---

### HOSPITAL PRACTICE.

### 104. Hôtel Dieu.

As the taste of the day is decidedly in favour of hospital cases and hospital reports, we shall endeavour to make this department of the Journal as extensive as we can. There are several *velocipede* vehicles running in the same line of road with ourselves, but as the wares they carry are more exclusively of English growth, we shall generally prefer articles of foreign manufacture; in other words, we shall make it our business to give a full account of the practice of the French hospitals. Hospital reporting is of much more ancient date abroad, and particularly in France, than here, and as far as the recording of practice is concerned, is carried on in a superior manner. The details are not so crimped as ours, and the reporter is evidently not so haunted by the dread of a *dele* from that *lethalis arundo*, which mars his jokes, cripples his sentences, and plays the deuce with half a quire of the most luminous (qy. voluminous?) "original remarks!" This is sad work, and it must be exceedingly annoying to the feelings of gentlemanly young men to find their "observations" cut short, especially when they remember that they are paid by the length of them! However, the affair is none of ours, and we leave it to the parties concerned, editors and reporters, to settle it between them.

### Abscess in the Iliac Region.

An abscess occasionally occurs in the right iliac region, apparently in the cellular tissue, which may open into the cæcum, vagina, or bladder. M. Dupuytren, who we verily believe sees every thing, has seen seven or eight examples of this disease, and the following is the description of the symptoms which it presents.

Pain, and circumscribed swelling more or less distinct in the iliac region, with feverishness, constipation, colic, nausea, hiccup, vomiting, all the symptoms, in fact, of an internal strangulation. There is a feeling of weight and uneasiness in the direction of the cæcum, and towards the anus, and after a little while fluctuation is more or less evident in the tumour. If the abscess bursts into the cæcum, vagina, or bladder, the tumour suddenly diminishes in size, and pus makes its appearance either in the stools, or in the urine, but sometimes, the abscess instead of taking any of these directions, travels across the parietes of the abdomen, and presents in the region of the kidney. This latter course is the most unfavourable; then that in which it opens into the bladder, whilst the most frequent and most favourable of all is its communicating with the cæcum.

The indications in the two latter cases, are to favour the issue of the pus, and allow the abscess to empty itself of its own accord. Strict regimen, perfect quiet, emollient drinks, and soothing local applications, are the proper means to be pursued, and under these the disease for the most part has a fortunate termination. A young man entered the Hôtel Dieu in September, in whom the pus was evacuated both from the bladder and intestine, and yet in spite of this double complication he did extremely well.

When the abscess passes across the walls of the abdomen, M. Dupuytren has never seen an instance of recovery; the surgeon, therefore, should not be in any hurry with his bistoury or lancet, but allow Nature to take her course, and if possible form a communication with the rectum. The opening, when it forms, being in the lumbar region, and the base of the abscess below, in the iliac region, the pus does not escape freely, but becomes contaminated by the mixture of air, proving an active cause of inflammation, and frequently inducing a fatal peritonitis or symptoms of re-absorption of matter. Under these circumstances, M. D. recommends placing the patient on his belly, in order to favour the exit of the pus as much as possible.

It may seem odd that, when the abscess opens into the rectum, the fæces and gas of the intestines do not escape into its cavity and produce unpleasant symptoms. Whatever may be the cause, whether the contraction of the abdominal muscles, the oblique and tortuous nature of the communication, or a portion of the gut itself falling down and acting the part of a valve, whether it be one or all of these circumstances it is impossible to say, but certainly the contents of the intestine do *not* escape from their proper cavity.

The affection is infinitely more common on the right side than on the left, which is evidently due to the anatomical relations of the parts. On the right side, the cæcum is comparatively little invested by the peritoneum, its posterior part is invested by a loose cellular membrane, and where it joins the small intestine (at the valve of the colon) a sudden contraction of the tube takes place, forming a kind of nest for the lodgement of irritating foreign bodies, as plumb-stones, cherry-stones, &c. On the left side the sigmoid flexure of the colon is of uniform calibre, and much more encircled by the peritoneum than the cæcum, whilst it is additionally strengthened by the aponeurosis of the iliacus internus muscle. These considerations amply account for the greater prevalence of the affection on the right side than on the left, as well as for the abscess, when it does form on the latter side, not opening into the sigmoid flexure of the colon, but passing down towards the groin, and becoming liable to be mistaken by a careless surgeon for inguinal or femoral hernia.

One or two cases are given where M. Dupuytren detected the disease, and gave a correct diagnosis where other practitioners had been deceived. In one case, that of the son of the Compte de B——, there had been for several days, feverishness, vomiting, pains and tumefaction on the right side, symptoms, which had led the medical man in attendance to pronounce the affection hepatitis. M. Dupuytren at that time had not seen much of the disease, but the circumscribed swelling in the iliac fossa, and the fluctuation made him suspect its existence. In the course of a few days a purulent discharge from the rectum confirmed the propriety of the diagnosis. —*Clinique.*

Any one who reads these clinical reports of M. Dupuytren's practice, must be convinced that he is an exceedingly shrewd observer of what is passing before his eyes. He is evidently a practical surgeon, and has very little indeed of that spirit of theorizing, which is the besetting sin of many of his countrymen. It is this which makes his "Clinique" so much *recherchée* in Paris, and gives a very great weight to what falls from his lips or his pen. A case very similar in many respects to the affection described by the Baron, occurred a few months ago at Guy's Hospital, and was talked of, and written of, in the Societies and Journals at the time.

*Case.* A young woman, of 19, was admitted into the clinical ward, under the care of Dr. Cholmely, with vomiting of yellow bile, relaxed bowels, small, wiry, and frequent pulse, hot and dry skin, and pain in the *right* iliac region, relieved by lying on the right side with the legs drawn up. Eight days previous to admission, she caught cold during the menstrual period, by washing in a damp cellar, when the catamenia suddenly disappeared, and four days afterwards she complained of acute pain in the right groin extending up to the iliac region with great tenderness on pressure. These symptoms led to the suspicion of hernia, but Mr. Callaway, who was called in, gave his opinion against the existence of that disease. During the three days preceding her admission, she was three times bled, and twenty leeches applied to the groin, and on her admission, the leeches were repeated, with calomel and opium every four hours, and clysters of castor oil. On the next day, she was rather better, but any attempt to lie on the left side produced severe dragging pain. *Treatment as before.* On the 12th, the pain and tenderness were severe, and 30 ozs. of blood were taken from the arm, when the catamenia re-appeared, but without affording relief. *Pill of calomel, opium, and tartarized antimony, with saline draught.* 13th. No better, pain and tenderness severe, with fulness in the inguinal and iliac regions. *Thirty leeches to the part—fomentation and poultice.* The pain, &c. in the iliac region continued, the pulse was rapid, the features sunken with nausea and sickness, but no constipation. Dr. Cholmely imagined, that there existed a deep-seated abscess between the muscles and peritoneum, and on the 15th, an incision was made a little above the anterior superior spinous process of the ilium, giving issue to a small quantity of sanious fluid, with fetid gas, and exposing a distinct cavity. Constant vomiting supervened, and on the morning of the 18th the girl expired.

*Dissection.* On the right side, the omentum adhered to the peritoneum, and formed a circumscribed cavity which contained some dark serous fluid; the peritoneum itself was black, but its surface was entire, and on cutting through it a large abscess was discovered, containing most offensive sanies. The psoas and iliacus internus were completely gangrenous, and the parts in the neighbourhood, viz. a portion of the right lobe of the liver, part of the tunics of the kidney, the posterior surface of the cæcum, and ascending portion of the colon, were all of a black hue. The appendix cæci was dark coloured, and its internal surface ulcerated.

This is a very good example of that variety of the disease which M. Dupuytren describes as originating in the iliac fossa, and passing up between the muscles and the peritoneum, in the direction of the kidney. The pain in the iliac region, the vomiting, the feverishness, and the fulness on that side very closely tally with the symptoms laid down by the French surgeon, whilst the unfortunate issue of the case completely verifies the gloomy prognosis which he draws in this dissection. Dr. Cholmely, it appears, has seen another case very similar to the present. A stout, healthy man, at Worthing, was attacked with the usual symptoms of nephritis and treated for that complaint. The disease then took a turn, and resembled enteritis, but in spite of the treatment had recourse to, he died in a fortnight from the day of attack. On examining the body, a large abscess was discovered behind the peritoneum, in the neighbourhood of the kidney and ascending colon, which had become gangrenous.—*Gaz.*

It is curious, that whilst the French surgeon recommends us not to meddle with the abscess, but, if possible, let it burst into the rectum or colon, the English doctor thinks the only chance of saving the patient is by making early incisions, and giving vent to the matter as soon as it is formed. Of course, in such a case, we can only repeat the Virgilian line.

"Non nostrum inter vos tantas componere lites."

But we must say, that we rather incline towards evacuating the pus, by an early incision.

Two or three years ago, we related a case similar in many respects to the abscess which M. Dupuytren describes as opening into the cæcum, but differing from it in this, that it also formed an opening by ulceration externally, allowing the fæcal matter to escape, and constituting that most disgusting malady, an artificial anus. In the case we allude to, the abscess formed in the glands of the groin, and not in the cellular tissue; the symptoms were very severe, and the termination fatal. There is a patient at present, in St. George's Hospital, whose case comes very near indeed to those which have occurred at the Hôtel Dieu, the abscess having apparently originated in the cellular membrane of the groin, and opened into the cæcum.

*Case.* Grace Harris, æt. 21, was admitted into St. George's Hospital, on the 4th of May, under the care of Mr. Brodie, with an artificial anus in the right groin, of which she gave the following account.

About a fortnight before Christmas, she received a severe kick in the groin, which gave her much pain at the time, and, in the course of a month, she began to be affected with pain and throbbing in the part, increased on walking, and obliging her to keep her room. She applied to Mr. Acret, of Torrington Street, who ordered her various medicines, and, subsequently, a blister, leeches, and fomentations. Four weeks, or thereabouts, prior to her admission, she first discovered swelling; it was fomented and poulticed, and, in a short time, a puncture was made by Mr. A. giving issue to nearly half a pint of the most abominably offensive pus. The abscess continued to discharge, and, at the expiration of a week from its being opened, she noticed fæcal matter in the poultice. From that time, the evacuation of the fæces took place, though not every day, through the opening in the groin, attended with a very relaxed state of the bowels, and much emaciation and debility. Since Christmas she had ceased to menstruate, and every two or three years, she had been subject to very severe attacks of what she denominated "liver complaint," which confined her to bed for several months.

On admission, there was observed a small sinuous opening, as large as a silver penny, about an inch and a half on the inside of the anterior superior spine of the os ilii. The integuments in the line of Poupart's ligament were inflamed, and bedewed with dirty-looking pus, of a fæcal odour, which constantly issued from the sinus. There was little or no pain either in the part or elsewhere, except on the passage of the fæces, when she felt a kind of gurgling and very disagreeable sensation in the groin. Her appearance was meagre, scrofulous, and hectic; she felt flushed and feverish in the evenings, and was harrassed, towards morning, with a troublesome dry cough, which she had suffered from for several years. The pulse was quick and wiry, the tongue reddish, the appetite voracious. She was ordered saline draughts with the sulphate of quinine, and, on the 19th, the discharge of fæces by the artificial anus having increased, rather than diminished, Mr. Brodie determined on laying open the sinus, and giving a freer vent to the discharge. This was done by introducing a director, and passing it for about an inch and a half downwards and inwards, in the line of Poupart's ligament, but above it, and as far as Mr. B. could judge, beneath the tendon of the external oblique. An incision was then made upon the extremity of the director, and the integuments, &c. divided along the groove to the opening of the sinus. On introducing his finger, Mr. Brodie thought he could pass it downwards, *under* Poupart's ligament, into the cavity of a considerable abscess. The wound was ordered to be dressed with lint, tow to absorb the discharge, oiled silk, and a solution of the chlorate of lime to be employed in order to destroy the effluvia. On the 20th, the bowels being very loose, the saline mixture was discontinued, and chalk mixture, with aromatic confection and tincture of opium substituted for it. Next day, however, the former medicine was resumed, and, on the 23d, the improvement was very perceptible, the discharge, both of pus and fæces, having lessened, and the general appearance being much improved. She went on improving until the 2d of June, when the bowels were confined, and the girl altogether unwell. The quinine, &c. was ordered to be omitted, and the bowels opened with calomel and colocynth. On the 6th, she was doing extremely well; the discharge had materially diminished in quantity, and, for the last six or seven days, had been completely unmixed with fæces.

In this case, there were none of those violent symptoms of peritoneal or intestinal inflammation which ushered in M. Dupuytren's cases, as well as those which occurred to Dr. Cholmely. The case, however, is clearly one, in which an abscess formed in the cellular membrane, lying in the "iliac fossa," and formed a communication with some part of the intestine, most probably the cæcum. That the opening into the gut is not of great dimensions, we think is probable, from the discharge of fæces through it being comparatively small in quantity, and occurring only every other day. The gurgling, too, and pain on the passage of the stool would seem to shew, that the communication is not a very free one. The progress of the case since the patient was received into the hospital has been hitherto extremely favourable, and there seems every reason to expect that she will ultimately do well.

---

### La Charité.

### 105. Diseases of the Testicle.

Case 1.—*Sarcocele of the Right Testicle—Amputation performed.* Auguste Shuilliers, æt. 27, of sallow, cachectic habit, entered the hospital of La Charité, on the 14th of June, 1827, under the care of M. Roux, for a disease in the right testicle, which had commenced in the early part of the preceding April. The testicle was about the size of one's double fist, the skin of the scrotum ulcerated in three places, and through the ulcerated openings funguses had sprung up, nearly an inch in diameter. There were some enlarged glands in the groin, and the spermatic cord was indurated and enlarged, almost as high as the inner ring, but no tumour could be distinguished in the abdomen. There were severe lancinating pains in the testicle and in the abdomen, whilst the stench from the former was most abominable.

After some hesitation, amputation of the testicle was performed, and, upon dissection, it was found that the tunica vaginalis, and tunica vaginalis reflexa, were intimately adherent, and destroyed in three several places to allow of the protrusion of the funguses mentioned above. No traces of the original structure, either of the testis or epididymis, remained, both being converted into a diseased mass, a compound of almost every kind of disorganization. At the lower part, there was a soft, whitish, diffluent matter, contained in two small cavities; in front, a reddish-brown substance; in the centre, a kind of nucleus, principally made up of that yellow tubercular matter, the result of chronic inflammation; and, at the posterior part, a white mass, hard in some places, half softened down in others, of which structure, also, the cord was composed.

If any one will be at the trouble of examining the cases of "sarcocele" which are reported and recorded, he will find that the majority of those which are really malignant, is made up of this anomalous structure, which fairly sets our classifiers of tumours at defiance. It may admit of dispute, whether, in such a condition of the parts, with a cord enlarged as high as the inner ring, and an unhealthy aspect, the patient was a fair subject for the operation. M. Roux thought he was, and for this reason, that, without it, his existence must soon be terminated by a loathsome malady; whereas, by its performance, he gains a few months of life, perhaps still more, and dies at last of a visceral disease, an incomparably preferable death. M. Roux believes that the immediate dangers of the operation are scarcely to be taken into the account, as they are few, and depend "on particular or local circumstances."* Thus, he instances the case of a patient, who, after castration, was attacked with severe erysipelas, causing sloughs on the scrotum and penis, and attributes this "accident" to a particular "constitution" reigning in the atmosphere at the time, and inducing a great disposition to erysipelas throughout the town, after very simple operations. There is much truth in this remark, and it is well known, that occasionally in our London hospitals, there is this disposition to erysipelatous inflammation so strongly shewn, that the surgeon's hands are almost tied. It has been observed in London, that this takes place after, or during, a long prevalence of Easterly winds, which might really be termed the English siroc, from their generally bad effects on the constitution of John Bull.

M. Roux makes some remarks on the operation itself which are worthy of notice. When he divides the cord, as in this case, high up, he makes the section of it gradually, taking up each vessel as it bleeds, because, at this height, the retraction is frequently very considerable when the cord is cut across at once. When the division is made below the outer ring, the retraction is of less consequence; but, even here; it takes place to some extent, if the diseased testicle has been very heavy, and M. Roux has observed that the spermatic vessels retract much more than the sheath in which they are bound up. M. Roux protests strongly against including the whole cord in a single ligature, instead of taking up the vessels separatim. Some surgeons declare that they never met with a bad accident from this practice, but Chopart relates the case of a man who died after this mode of operating, with acute inflammation and suppuration, extending to the loins, and tetanus followed it not long ago, in one of the Parisian hospitals. With regard to the glands which were enlarged in the groin of this patient, as they appeared stationary, and but little swollen, M. Roux was disposed to consider them as merely affected from sympathy with the irritation of the scrotum. If they had been circumscribed, and of great size, he would have extirpated them, just as enlarged glands in the axilla are extirpated with the cancerous mamma.—*Clinique.*

The sequel of the case is not detailed; but, with so palpably a malignant disease, the hopes of ultimate cure are slight indeed! Generally speaking, after amputation of the testicle for an affection of this kind, the lumbar glands become affected, and a tumour is noticed in the abdomen, which rapidly increases. A good instance of this occurred not long ago at St. George's Hospital.

Case 2.—*Anomalous Disease of the Left Testicle—Extirpation—Appearance of a Tumour in the Abdomen.* W. Westbrook, a hale-looking countryman, was admitted into St. George's Hospital, Feb. 27th, with an oval tumour in the scrotum, about the size of a small melon. The tumour was hard and unyielding, except at the upper part, where there was a distinct projection, around which was a degree of softness and bogginess, not exactly amounting to fluctuation. The testicle was heavy, not very painful on pressure, or, indeed, at any time; cord apparently enlarged above the outer ring; veins of the scrotum distended; a good deal of pain in the loins, particularly at night; costive bowels. The other testicle had not descended through the outer ring. Two years previously he had received some injury to the part, which, for a few

* We have seen one patient die of acute peritoneal inflammation after the operation, and there are several cases on record of tetanus supervening, even when the vessels have been separately tied.

days gave him considerable inconvenience. The swelling appeared two months before his admission, after a severe day's work, and was attended with a great deal of pain in the testicle. He applied to a surgeon, who bled and leeched him, with much relief, but on resuming his employment, the symptoms re-appeared, and from that time went on progressively increasing.

After a little purging, the patient was put under the influence of mercury, whilst, at the same time, leeches were frequently applied to the scrotum. These means were quite ineffectual, and the health was beginning to suffer considerably, when he was ordered decoctum sarsæ with spt. ammoniæ aromaticus, and on the 27th of March, extirpation was performed by Mr. Rose. On making a section of the tumour, it was found to be composed of medullary matter, portions of scirrhus, and serous cysts upon the surface, resembling what are commonly called hydatids, with here and there small specks of the yellow tubercular substance.

For the first 48 hours after the operation, there was a good deal of irritability, with some diffuse inflammation in the direction of the cord. Under the use of saline draughts, with antimony, these bad symptoms disappeared, his appearance improved, and the wound went on slowly healing. In the course of a short time, however, he again began to look miserably sallow, and to complain of flatulence, whilst, at the same time, there was discovered in the abdomen, a little above and to the left of the umbilicus, a tumour as large as a melon, and attended with a little pain on pressure. This tumour increased in size, in spite of the tincture of iodine, and another was also felt in the left iliac region, apparently a process or continuation of the former. On the 6th of May, the poor fellow was directed to try his native country air, with permission to return to the Hospital whenever he should think proper.

This case is not very dissimilar to M. Roux's. There was found, on dissection, the same mixture of morbid growths, scirrhus, medullary matter, yellow cheesy structure, serous cysts. The disease was not attended in this instance, with the pain and suffering which accompanied the former, but then it had not proceeded near so far, as there was no ulceration of the scrotum, no fungous growth. We should remark, too, that the appearance of induration in the cord, in this case, before amputation, was deceptive, for it was found, during the operation, that the cord was pretty sound, though a *process* extended over it from the tumour, quite through the outer ring. The sudden appearance of so large a tumour in the abdomen, immediately after the operation, is but a bad augury for the future fate of the unfortunate patient; it serves to shew how extremely rapid the progress of internal glandular disease is, when the external has been *cut* short.

In a case of fungus hæmatodes of the testicle, reported in the Medical Gazette, we find it stated, that in several cases of that disease, which Mr. Brodie has witnessed, the patients have died, after the operation, with true fungus hæmatodes of the lungs. Such persons are suddenly attacked with symptoms resembling violent inflammation of the pleura, and, to the astonishment of their medical attendants, are rapidly cut off. On dissection, the lungs are found affected with the above disease, and serum and lymph effused into the cavity of the chest.

It is well known, that the obscurity attending the diagnosis of many of the diseases of the testicle, is such, that it has been publicly recommended by Sir Astley Cooper, and many other surgeons, to plunge a lancet or trocar into the tumour, before having recourse to amputation, lest it should turn out to be merely a hydrocele or hæmatocele. They tell indeed, a very pleasant but rather scandalous story of Sir Astley, at the Hôtel Dieu. The worthy Baronet, as every body knows, was deservedly a "lion" at the French Hospitals, and being in the operating theatre of the Hôtel Dieu, with a great concourse of students assembled, a patient was brought in with a tumour in the scrotum. Before removing it, M. Dupuytren handed the patient to Sir Astley, to ascertain that all was right. After a slight examination, Sir Astley handed the patient back to M. Dupuytren, with the assurance that all *was* right, when the sly Frenchman immediately punctured the tunica vaginalis, and evacuated, we cannot say how much, fluid! A buz, of course, went round, at the expense of the "Grand Chirurgien

Anglais," which we need scarcely say, he bore with his accustomed good humour.

We are sure we shall be forgiven for taking this liberty with the reputation of one who can afford to lose so much, but we were led to relate the piece of scandal, from observing a case in which M. Dupuytren was deceived in the same way, though not by a brother surgeon.

*Case.* 3. A man of 28 or 30 years of age, was admitted into the Hôtel Dieu, on the 27th June, with a considerable enlargement of the left testicle, which appeared fifteen years previously, after a blow received in mounting a horse. Since that, he had several times hurt the part, and increased the enlargement which proceeded very slowly. When admitted, the testicle was the size of a double fist, hard to the touch, and without fluctuation, except at its upper part. The scrotum covering it was tense, red, and inflamed; the tumour was not transparent; and there were violent lancinating pains in it, extending along the spermatic cords to the groins, and lumbar regions.

As the disease had existed so long, M. Dupuytren was not inclined to consider it either venereal or scrofulous, whilst the total want of transparency seemed to shew that it was not a simple hydrocele. Cases, however, occasionally occur, in which the hydrocele is complicated with a cartilaginous thickening of the tunica vaginalis, deceiving the surgeon and leading him to extirpate the testicle "to the great scandal of the assistants," and we should say, of himself. Accordingly, though firmly of opinion that the present tumour was malignant, M. Dupuytren determined on making an incision into the tunica vaginalis, and then, if neither water nor blood should flow, to proceed with the operation of castration. The incision was made, the tunica vaginalis found to be filled with fluid, and the membrane itself so thick and solid, as to resemble the half of a cocoa-nut! The wound was dressed with lint, granulations sprang from the interior of the scrotum, the remainder of the thickened tunica vaginalis came away, and thus the patient was spared a testicle, and the surgeon a very mortifying blunder.

### ST. GEORGE'S HOSPITAL.

### 106. RUPTURE OF THE DIAPHRAGM AND VENA CAVA ASCENDENS.

A boy was taking care of a horse and gig, when the horse ran off and knocked him down, the wheel of the gig passing over his body, whilst, at the same time, a lamp-post fell also, but whether upon the boy could not be ascertained. He was lifted up and conveyed to a surgeon's in the neighbourhood, who immediately opened a vein in the arm, and contrived to abstract some six or seven ounces of blood. Being apparently little the better, he was carried to St. George's Hospital, and admitted, about 2 P. M. in a state of the most complete prostration, the whole body pale and cold, the pulse at the wrist to be felt, and that was all. In ten minutes after his arrival he expired.

On opening the chest, the left cavity of the pleura was filled with a quart, or more, of dark venous looking blood. The diaphragm on that side was rent across, and the spleen, literally torn into tatters, had been driven through the lacerated opening, and lay within the thorax. The stomach was still in the abdomen and free from injury, with the exception of the vasa brevia, which were broken up, and blood very extensively effused beneath its peritoneal coat. The splenic artery and vein were sound and still attached to a ragged portion of the spleen which remained in the abdomen. On lifting up the liver, a laceration of considerable size was discovered in the vena cava, where it passes through the tendinous opening of the diaphragm, and is joined by the hepatic veins.

The reporter remarks upon the case that, "at the instant the wheel passed across the belly, the diaphragm must have been powerfully drawn downwards in inspiration, and the abdominal viscera thus subjected to great compression, in consequence of which the spleen was driven through the rigid and contracted diaphragm. That the violence with which the spleen was forced into the thorax was excessive, is abundantly evident from the complete *mash* in which it was, and indeed we do not believe it possible for the above injury to happen unless the diaphragm were in contraction at the time. The rupture of the cava is more difficult to explain, for it lies not in a muscular but tendinous opening, and is therefore comparatively free from the effects of compression.

As for the bleeding which was practised, it is difficult to conceive that there could have been the slightest indication for it, unless it were upon the principle that a boy had been run over, and therefore *must* be bled."—*Gazette*.

A very similar case occurred at the Hospital La Charité.

A man, about 40 years of age, fell from a height of thirty feet, and died in seven hours from the occurrence of the accident. On dissection, a considerable quantity of blood was found in the cavity of the abdomen, the liver was torn in several places, the gall-bladder also torn, empty, and collapsed. The stomach was not in the abdomen, but on opening the chest, it was found in the left cavity of the pleura, greatly distended, and filled with half-digested food. The lung was uninjured, but there was a very extensive laceration of the muscular portion of the diaphragm, through which the stomach had been forced into the thorax. Several ribs were broken on the left side.—*Clinique*.

---

### 107. Influence of Mercurialization on the Plague.

M. Moreau de Jones has communicated the following piece of information to the Royal Academy of Sciences of Paris. An Ionian boat having had intercourse with a Turkish vessel, the master of the boat became affected with plague, and in this state arrived at Cephalonia, where he and the boat's crew were put into the Lazaretto. The English surgeon of the Lazaret, conceiving that the crew of the boat had received the contagion, put them all, to the number of twelve, on an active course of mercury, internal as well as external. The whole of the boat's crew were successively attacked with plague, but with a remarkable difference in the degree of intensity. The master, and one of the crew who had not experienced any sensible effect from the mercury, died of the disease. The other ten became salivated, and had the disease in a mitigated form. They all recovered.

This remarkable event, says M. De Jones, naturally leads us to conclude that the escape of ten out of twelve was owing to the influence of the mercury—a conclusion strengthened by the fact that the two who died could not be brought under mercurial ptyalism. He observes that mercury was administered in the plague which desolated Malta in 1813; but it was given during the existence of the disease with the hope of *curing* it, and not as a *prophylactic*—which makes a great difference in the result. M. De Jones recommends mercurial frictions to be used in preference to the internal use of mercury, as a preventive, the former not being liable to objection on the score of irritating the stomach and bowels.

That fevers and other diseases have sometimes invaded those who were in a state of ptyalism we know; but, at the same time, we have seen sufficient facts to convince us that mercurialization exerts a considerable prophylactic influence on the human constitution, when febrific causes, as contagion, &c. are diffused through the atmosphere. As it is likely that our ships shall frequently come into contact with infected vessels, during the approaching operations in the Mediterranean, the above piece of information may be worthy of notice.

---

### 108. On the Use of Stramonium. By Drs. Wendt and Elliotson.

In the 24th volume of Rust's Magazine, Professor Wendt has published some curious observations on the medicinal and physiological effects of stramonium, which we shall briefly notice. According to this writer, the action of stramonium is analogous to that of belladonna, excepting that it has much less influence on the circulation, and exerts a specific power over the genital organs. Thus, he informs us that, given in full doses, to a man in good health, it excites the venereal appetite; but if administered to an individual labouring under morbid excitement of the genital system, it calms that excitement. The medicine, he observes, is indicated in all cases of irritation, or even phlegmasia, especially where there is an attendant train of nervous symptoms. He recommends it in nymphomania, epilepsy from onanism or other excitement of the genital organs—inflammation of the spinal marrow—diaphragmitis—carditis, &c. first taking care to reduce inflammatory action to a proper extent by venesection and other modes of depletion. The preparation Professor W. recommends, is a tincture made by infusing two ounces of the dried seeds in eight ounces of malaga wine, and one ounce of alcohol. The dose is five, ten, or twelve drops, every two or three hours, for an adult.

In a late number of our cotemporary, the MEDICAL GAZETTE, we observe some reports from St. Thomas's Hospital, where Dr. Elliotson has been exhibiting the extract of stramonium for the relief of periodical pains, or, in other words, neuralgic affections, which are often as distressing to the patient, as they are baffling to the practitioner.

The first case was that of a female who had excruciating pain of the left side of the face and neck, occurring every afternoon about five o'clock, and continuing four or five hours. The intervals were entirely free from pain. The health was otherwise pretty good. A grain of the extract of stramonium was given at 4 o'clock every day. By the third day the paroxysm was mitigated. The dose was increased one half. In ten or twelve days the paroxysms were stopped.

As neither bark nor arsenic was employed in this case, we cannot say whether they would not have been as effectual as the stramonium.

The second case was complicated with some pectoral and hepatic complaints, for which mercury was given, in conjunction with the stramonium. By these means the periodical pains and the other affections were removed, ptyalism having been induced.*

The third case was of a different character. The patient was also a man, aged 47, who was affected with severe gastrodynia thrice every day—at ten in the forenoon—five in the afternoon—and at midnight. His appetite was good, and he had no tenderness in the epigastrium. He was a painter by trade, and had had this complaint, more or less, for two years. The sulphate of quinine was here employed fairly; and though it gave temporary relief, it completely failed in curing the disease. Having given some hints that he had had tape-worm two or three years previously, the oil of turpentine was exhibited in doses of an ounce night and morning. By this remedy he was cured in a few days, although no tape-worm was ejected. We have no doubt that the gastrodynia, in this case, was caused by the emanations of lead—in fact, that it was a grade of colica pictonum.

We have recently met with a violent case of colica pictonum, produced by the superacetate of lead taken in the dose of only one grain, with an equal quantity of opium, for the space of about three weeks. The complaint for which this remedy had been given was seminal emissions, and was apparently cured, when the symptoms of colica pictonum supervened. They resisted all means till ptyalism was raised, when the bowels became free, and the pain about the umbilicus, attended with porraceous vomiting, ceased. We have often given the superacetate in larger quantities and for a longer period before, but never saw colica pictonum induced by that remedy.

* Dr. Elliotson informs us that gargles of the solution of chlorate of soda very soon put an end to mercurial salivation in his practice.—*Ed.*

---

109. MEMOIRE SUR LE RAMOLLISSEMENT DE L'UTERUS. MEMOIR ON MOLLESCENCE (MORBID SOFTENING) OF THE UTERUS. By S.G. LUROTH, M.D. [HOSP. LA MATERNITÉ.]

It is only of late years that MOLLESCENCE, or morbid softening of the living structures, was discovered to be one of the most important, and, unfortunately, one of the most common structural lesions to which the human frame is subject. The brain, the lungs, the spinal marrow, the nerves, the muscles, the bones, the heart, and other parts, have been found in this pathological condition, and have been the subjects of accurate investigation. The uterus is the organ to which we are now to direct our attention.

The MOLLESCENCE of the womb is more frequently partial than general. It more commonly occupies the internal surface, and the cervix—though occasionally it is found penetrating through the entire substance of the organ. The MOLLESCENCE presents several *degrees*, blending insensibly into each other. In the first degree, the parts are simply softened or very flaccid, generally with serous, or sero-sanguineous infiltration into the interstices. An example of this kind was observed by our author in the Hospice de la Maternité, in the month of March, 1827. A young and strong female had been carried off by puerperal fever, a few days after delivery. On dissection, not only the uterus, which had a large empty bag, but all the other organs, especially those of a muscular structure, as the heart, were in a state of extreme flaccidity. The tissue of the uterus was infiltrated with serosity, and very lacerable. Internally it was lined with a dark-coloured viscid coating, exhaling a putrid odour. The ovaria were softened, flaccid, and infiltrated. The heart was in a similar condition. There are not wanting examples of this kind in works on puerperal fever.

In the second degree, the structure of the uterus is still farther altered. It will scarcely bear handling, without reduction into a pultaceous mass. The following is an example, observed at the Hospice de la Maternité, under the care of Professor Deneux.

A female, aged 27 years, of good constitution, and previously healthy, was safely delivered of her second child, 4th April, 1827, after a labour of seven hours. She complained of a pain in her side the same day, and was bled, both generally and locally; but some symptoms of pulmonic affection continued till the 17th of the same month, when she complained, for the first time, of burning heat in her throat. The night was passed in great agitation, and, on the following day, the tongue was observed to be swelled, and an erysipelatous eruption covered the neck and shoulders. As there were some signs of gastric derangement, an emetic was prescribed, and an oily purgative. On the 19th, the patient complained of great general debility, but no local pain. Having exposed herself to cold by throwing off the bed-clothes, the erysipelas disappeared rather suddenly, and was succeeded by diarrhœa, urgent thirst, and cough. 22nd. Cephalalgia was added. 23rd and 24th. Shewed symptoms of low fever; but without any pain or tenderness of the abdomen. She lingered till the 26th, when she expired, never having complained of pain in the abdomen or uterine region.

*Dissection.* The arachnoid was opake—many red points in the brain when sliced—no effusion in the ventricles. There was some yellowish serum on the thoracic cavities—lungs sound—heart flaccid. There was some yellowish effusion in the peritoneal cavity, but the peritoneum itself was healthy. The mucous membrane of the cæcum and colon was intensely inflamed—liver enlarged and softened—the uterus was so soft, that it would scarcely bear handling, especially its anterior parietes.

In two cases of puerperal fever, our author observed a similar mollescence of the uterus ; and cases are quoted from Lippich and Nauman, shewing the same condition.

In the third degree of uterine mollescence, the disorganization amounts to almost a liquefaction, or reduction of the viscus to an inorganic pulp. Generally, this state is only partial—life not continuing till the whole organ is so changed—especially in acute cases. For the most part, the stomach, heart, or other viscera, partake in these mollescences. The tissue thus softened, sometimes preserves its natural colour—at other times it is pale. The parietes of the uterus are more frequently in a state of atrophy than hypertrophy, when they are morbidly softened.

The symptoms of this uterine mollescence are very vague, and but little known—especially those attendant on the invasion of the disease. A sense of weight, or constraint in the pelvis—dull pain in the hypogastrium, augmented by pressure—uterine hæmorrhage—suppression of the lochia (if in the period of accouchement)—febrile exacerbations, &c. are the usual accompaniments of this disease; together with a remarkable prostration of the mental and physical powers, and a presentiment, on the part of the patient, that death will ensue. Our author thinks—and the conjecture is rational—that, if this disease be going on during utero-gestation, there will be slow and laborious parturition, with probability of a dead fœtus, uterine hæmorrhage, and other accidents attendant on bad labours. It is also not improbable, that mollescence of a portion of uterus may be very accessory to that dreadful occurrence, laceration of the organ. This last supposition, indeed, is nearly converted into a certainty, by the cases of ruptured uterus put upon record by various authors. The rupture of other organs also, as the heart and stomach, in cases of mollescence of these parietes, is favourable to this supposition.

The progress of mollescence of the uterus, is sometimes acute, sometimes chronic. The duration, of course, is very various. It may continue for many years, if the affection be partial, and if nothing occurs to hurry forward the disease. Without being able to say any thing decisive as to its comparative frequency, our author thinks, that it is a malady by no means very rare. The proximate cause is doubtful—it is not always the same. The mollescence is sometimes a primitive affection—sometimes the effect of other diseases, as of inflammation. In the first instance, it may be owing to a kind of defective nutrition—in short, it may be a kind of atrophy of the organ, unaccompanied by any super-irritation or excitation. There is little doubt, however, that this mode of production is infinitely more rare than that which results from inflammatory action in the part, acute or chronic. Our author is also of opinion, that there is another cause for this mollescence of the uterus—and that is, a putrid or depraved state of the blood itself. Many cases which occurred to him at the MATERNITÉ, are in support of this doctrine; but, at present, he declines entering farther on this path of investigation.

The *prognosis* is unfavourable—but more or less so, according to the degree of intensity, and extent of the disease. The *diagnosis* is very difficult, and must be gathered, if possible, from the few symptoms already described. In most cases, the disease is only recognized after death. This, however, was the case in many other diseases, now well known by living indications, but formerly undistinguishable, from want of investigation. The same observations will apply, no doubt, to the treatment. It must be purely *symptomatical*—that is, the actual phenomena present must be attended to, and combated, if practicable. In some cases the disease, when situated about the os, or cervix uteri, can be recognized by manual examination. It is then for us to determine, by the existing symptoms, whether it is of an inflammatory nature, or the effect of atrophy—and act accordingly.—REPERTOIRE.

### 110. DR. HOSACK ON REMOVAL OF THE TONSILS.

The removal of enlarged tonsils is an operation of frequent occurrence, and many contrivances have been invented for facilitating the extirpation, and preventing hæmorrhage. These contrivances we need not enumerate—they were all attended with pain and difficulty. The following is Dr. Hosack's plan, which has simplicity and celerity in its favour—and, we believe, is equally safe as those which are much more harrassing to the patient, and embarrassing to the practitioner.

"Having had an opportunity, during my late residence in France, of frequently witnessing the successful removal of the tonsils by the knife, and having, since my return, performed that operation in six instances with the same favourable results, I avail myself of the opportunity afforded me through the medium of this journal to call the attention of the profession to the manner of conducting this operation, and the advantages it posses-ses over every other mode of removing the diseased tonsils—nothing more is necessary than to pass a hook through the body of the enlarged gland, and to raise it from its bed between the arches of the palate, then to pass a probe-pointed bistoury under its base, and with one stroke to sever it from its connexion: the hæmorrhage never exceeds two ounces, and is always to be arrested by a draught of cold water.*"

This operation is not of modern date. Celsus and Paulus Eginetus recommended this plan, which was objected to by Fabricius Hildanus as hazardous, and then the actual cautery came into use. The ligature succeeded, and is still, perhaps, the measure most usually employed. Dupuytren, Roux, Vacca, and many eminent surgeons on the Continent, with Physick, Hosack, and others, in America, prefer excision.

### LA PITIÉ.

### 111. EPIDEMIC ERYSIPELAS—TREATMENT BY LEECHES AND BLISTERS.

Since the 24th of April last, erysipelas has been extremely prevalent in the Hospital of La Pitié, following the most trifling operations, as the application of a seton, or a leech-bite.

*Case* 1. A man, æt. 30, of a bilious temperament, had a seton in the nucha, for a

* American Journal of the Medical Sciences.

very obstinate ophthalmia. An erysipelatous blush immediately appeared on the back of the neck, with hot and dry skin, quick pulse, tongue white in the centre, and red at the edges. Forty leeches were applied to the epigastrium and the seton taken out, but pain in the head supervened, and on the 2d of May the scalp was swollen, red, and painful, the erysipelas had attacked the face, the patient was delirious during the night, the tongue was very dry. *Thirty leeches to the occiput.*

*May 3d.* The erysipelas has spread over the whole face, the features are sunken, the pulse small, the tongue not red, but remarkably dry, the patient stupid and delirious. *Sinapisms to the legs—blisters to the thighs.* On the next day the delirium disappeared, the symptoms gradually subsided, and on the 10th of May the man was discharged cured.

The next case was similar in many respects to the preceding, but the termination was unfortunate.

*Case* 2. An old man, of 64, was admitted into the hospital for an enlargement of the parotid gland, which was treated at first by compression, and subsequently by the application of leeches. On the 25th of April, erysipelas appeared upon the cheek, the tongue was red and dry, the skin hot, the pulse rapid. Venesection in the arm, and the application of a considerable number of leeches to the epigastrium, were the means employed, but in spite of them delirium supervened, the pulse fell, the tongue got dryer, the erysipelas spread over the face, and terminated in phlyctenæ, and on the 2d of May, seven days from the first appearance of the disease, the patient sank.

On dissection, the membranes of the brain were found inflamed, and the cerebrum itself of firmer consistence than it should be, and covered with drops of blood. The stomach and intestines were quite sound.

It appears that this patient had not only the disease, but himself to contend against, for he procured quantities of wine and food without the knowledge of the surgeons, and refused to submit to the regimen prescribed. Under these circumstances, it is no great wonder that depletion should have failed in arresting the disease.

In the succeeding cases, blisters were employed as well as leeches.

*Case* 3. A boy of 16, affected with the morbus coxarius, had a certain number of leeches applied to the hip, which were followed on the next day by erysipelas. The tongue was red and dry, the skin hot, the epigastrium tender to the touch. Thirty-five leeches were applied to the latter region, when the erysipelas attacked the thigh. A blister was applied to the inflamed part, but could not be got to rise, and of course had no effect. The erysipelas spread to the leg, and was assailed by another blister, which rose well, and routed the enemy at last.

The above was evidently a common case of erysipelas which would have been treated in this country by salines and low living. We never had an opportunity of witnessing the employment of blistering in this disease, but we should conceive that so irritating an application to an inflamed part, must be extremely painful and moreover unnecessary in ordinary cases. That blisters have but little effect, if not a positively bad one, in severe cases, the following will prove.

*Case* 4. A woman of forty-five, whose constitution was worn out by the sufferings of cancer, had the breast removed on the 25th of April. On the next day erysipelas appeared around the wound. Forty leeches were applied, and there being some symptoms of gastritis, twenty more were ordered to the epigastrium. The patient felt extremely weak; there was no thirst, but the skin, notwithstanding, was burning hot; the tongue was dry, but not preternaturally red, and there was pain experienced in the left side of the chest. *Leeches to the thorax.* The erysipelas "travelled" to the right shoulder, and was attacked there by a blister, which seems to have proved of service; for on the 2d of May, we find that the disease had nearly disappeared. In the course of a short time the erysipelas returned, and took possession of the whole of the right side of the trunk, was treated by the application of a second blister, and on the 9th, had once more disappeared, leaving however some diarrhœa, and pain in the abdomen. *Fifteen leeches to the anus.*

10*th.* A most unfortunate change has taken place; there is great anxiety, the aspect cadaverous, the pulse "miserable," the tongue dry, but still not red, the mucous membrane of the mouth entirely discoloured. Ten leeches were applied to the umbilical region, but without avail, for on the morning of the 11th the patient sank.

Evidences of inflammation of the pleura on the left side, and of peritonitis were discovered on dissection. There was some discolouration of the mucous membrane of the stomach, but no trace whatever of inflammation in the intestines.

It may admit of doubt whether or not the pleurisy and inflammation of the peritoneum which occurred in this poor woman, were aggravated by the blistering on the surface, for such a means would be not unlikely to repel a cutaneous inflammation, and throw it on a vital organ. We remember to have seen a case, where the breast was amputated for cancer in a young and apparently healthy woman, and where erysipelas attacked the wound. The patient died, and on dissection a considerable quantity of pus was found in that side of the chest, apparently shewing that a considerable disposition exists for inflammation to extend from the walls of the thorax without, to the serous membrane which lines its cavity within. Facts of this kind are of use, as they tend to put the practitioner on his guard against employing any measure which may tend to favour this metastasis or disposition, whatever it may be, and throw the onus of the inflammation on the pleura or the lungs.

Baron Larrey is famous for the actual cautery. We remember when this worthy old surgeon was in London several years ago, he was shewn a patient with a syphilitic ulcer of the pharynx. The case was obstinate, and the patient had been for some time under treatment, but immediately the Baron saw him he recommended the actual cautery as a certain cure, declaring that the man would never feel it. The hint was not acted on. At the Hôpital de la Garde Royale, they are in the habit of treating erysipelas by this rather disagreeable application, determined, we suppose, to "put out" one fire (St. Anthony's) by another.

*Case* 5. A young soldier, of lymphatic temperament, had a tumour of considerable size in the right axilla, in the centre of which fluctuation appeared, whilst a good deal of chronic induration remained around. Potassa fusa was applied to the fluctuating part, and the slough which formed, cut into. Erysipelas, however, supervened, and extended with great rapidity over a great portion of the thorax. The surface was lightly touched, at several points, with a cautery, at a dull red heat. The erysipelas ceased to extend, and in a few days had completely disappeared.

Such treatment as the above might succeed in a robust young soldier, but we question whether it would not often be an overmatch for the enfeebled constitutions of many hospital patients. In common cases of erysipelas, "heroic" treatment of any kind, whether it be the actual cautery or twelve-inch incisions, is not only unnecessary but cruelly severe, for salines and low living are sufficient. When the erysipelas is of a florid and phlegmonous hue, the pulse rapid and full, the patient of an inflammatory diathesis, incisions are often of the greatest service, as they tend to relieve the tension of the vessels, unload the part of blood, and give vent to matter, which forms in the sub-cutaneous cellular membrane, long before the integuments themselves give way.

*Case* 6. A young man, of florid aspect, was admitted lately into St. George's Hospital, with erysipelas of the right leg, extending to the knee, and following a "blister" on the foot, received in walking. He was ordered a poultice, cold lotion, and salines with antimony, but on the 4th day from his admission, he was slightly delirious, with quick pulse, and brown tongue. The erysipelas appeared upon the face, and the whole leg was of an uniformly scarlet tint, the cuticle being separated in extensive patches. Very free incisions were made by Mr. Brodie in different directions down to the subcutaneous cellular membrane, which was sloughy and infiltrated with pus.

The florid tint of the limb subsided, the bad symptoms went off, and in the course of a few days the erysipelas had disappeared. Suppuration ensued in the cellular membrane, which was treated by poultices, &c. and in a short time the patient is to leave the hospital.

---

### 112. HOSPITAL REPORTS—NEW MEDICAL JOURNAL.

Just as this Number was closing, we received the prospectus of a new medical journal, entitled the "MIDLAND MEDICAL and SURGICAL REPORTER," to be dedicated chiefly to the publication of authentic hospital reports, collected from the provincial hospitals. The following extract will convey some idea of the objects and plan of this new candidate for public favour.

"We live in a busy age. In no former time, has so much intellectual activity been displayed. The cultivators of medical science have eminently distinguished themselves in the general pursuit of knowledge. The proof of this assertion will be found in the medical annals of the last thirty years. This advancement of our profession has been accompanied by a caution, and zeal, in conducting its periodical journals, that have no parallel in the history of our art. No circumstance has tended more to improve it in France, Germany, and Italy, than the manner in which the local History of Medicine has been given to the Public. There is scarcely a district of any extent in those countries without a periodical Medical Record. In this kingdom, however, great as have been the efforts thus made, they have almost exclusively been confined to the three Capitals, London, Edinburgh, and Dublin. The provinces have had no share in this pursuit, or at least only so far as the journals of the Capitals have derived support from the contributions of country correspondents. In reflecting on the state of medical science, and on the aid which it has derived from the opportunities of observation afforded by congregating the sick in hospitals, it has often struck us with surprise, that so little should be known of the many interesting cases that must have occurred in provincial Hospitals. If, from the establishment of these Institutions, faithful records of the valuable cases that have occurred in them had been handed down to us, our present limited knowledge of Morbid Anatomy, the only correct elucidator of Disease, would have been greatly extended. We know well that great difficulties must present themselves in effecting an object so desirable, as making public these instructive cases. The daily anxieties and fatigues to which those in general are subject, who have the professional care of these Institutions, are such as to afford little time for literary pursuits, and appear to present an almost insurmountable barrier to such an undertaking. It is not, however, to be denied, that by zeal and industry much may be effected, and the paramount importance of their object will induce the Editors of the Midland Reporter to endeavour, by every possible means, to make a faithful report of all matters of interest, occurring in the several Institutions to which they are attached. Neither will their means of affording information end here. They reside in the centre of a populous district, where facts of importance are doubtless not unfrequent occurrences, and they have made such arrangements as will ensure an early notice of them. It is also the intention of the Editors to commence each number of the Reporter by an Essay on some Medical or Surgical subject. To carry on this part of the undertaking, they must be, in some measure, dependent on their friends, and for this purpose they earnestly solicit their prompt and steady support.

"As the Reporter is to be strictly a record of facts, no Reviews will find a place in it; but every thing that can in the slightest degree tend to enrich the topography of the district, will be readily inserted, more especially observations Statistical, Botanical, Geological, and Meteorological.

"The growing interest taken by the Profession in Medico-legal Science, will induce the Editors to use their best efforts to add to the hitherto scanty supply of facts in this important branch of knowledge. This object, they conceive, will be best attained, by giving a succinct account of the most essential particulars of all interesting cases of this nature, that may occur in the Judicial Courts within their district. The intention, in short, of the Editors, will be to present such an account of all important Medical and Physical occurrences, that come under their observation, as will enable the Profession to ascertain the diseases of the neighbourhood, and trace the connection, as far as the present imperfect state of the art will permit, with any peculiarity in the soil or climate, or in the habitations and engagements of the people.

"It is manifest, that if publications, on the above principles, be ably conducted in different districts, such a History of Endemic Disease will be made, as shall indicate the varying modifications of disease, in different situations, and at various periods. Thus will be obtained, by the combined exertions of many, and with little individual labour, an efficient means of investigating the genuine character of recurring epidemics, and disease in general.

"The Editors by no means confine their call for assistance to the Members of the Medical profession; many individuals, not of that Faculty, may eminently contribute to the great object they have in view; Gentlemen who reside much in the country, Men of general Science, and especially the Parochial Clergy, by the communication of facts in Natural History, local descriptions, the Flora of their districts, deviations from the ordinary course of nature, the prevalence of empiricism, &c. will materially assist their design.

"All Letters and Communications addressed (under cover) to the Editors, free of expense, at the Office of the Worcester Journal, will receive immediate attention. The first Number of the Reporter will be published on the first of August, price 2s. 6d. and the succeeding Numbers Quarterly. Gentlemen disposed to countenance the work, are requested to signify their intention to the publishers."

No possible doubt can be entertained, that the provincial hospitals afford an ample field for a quarterly journal of Hospital Reports; and we applaud the principle which stimulates our country brethren to the undertaking. We shall not fail to give every encouragement in our power to the work, and most sincerely do we wish it success. Every thing, however, will depend on the talents of those who are embarked in the enterprize, and the zeal of those who are to furnish the materials of the work. We would recommend the plan pursued in Paris—that of selecting an intelligent pupil, in each county hospital, to draw up the reports, making him responsible for their fidelity and correctness.

### 113. ANGINA PECTORIS.—PROGRESS OF PATHOLOGY.

At one of the late meetings of the College of Physicians, an interesting case of angina pectoris was read by Dr. Warren, of which we shall give a short account, and on which we shall take the liberty of making a few comments.

The patient was an elderly lady, who had enjoyed good health up to the accession of the present complaint, which terminated fatally in the course of ten or twelve weeks. The malady commenced with a sense of distressing pain under the superior portion of the sternum, which gradually increased, and extended, first to the shoulders, and afterwards down both arms to the wrists. The patient herself thought the pain was rheumatic; but its intensity soon became greatly increased, especially when she went up stairs, or took any considerable exercise. The attacks came on in paroxysms, consisting of acute and burning pain under the sternum, shooting thence to the spine—up along the neck—and down along the arms. Irregularity of breathing, great anxiety, and dread of instant death, attended these paroxysms.

When Dr. W. first saw the patient, she was recovering from a most violent attack; and was sitting in a chair, with her shoulders raised, and "breathing only with the lower part of the thorax—not moving the upper ribs." The pulse was about 100, soft and regular. Some relief was afforded by the abstraction of a small quantity of blood; but the paroxysms continued to recur, and one night, while turning herself in bed, she suddenly expired.

*Dissection.* No fluid in the cavities of the pleura—a small quantity in the pericardium—lungs sound—"heart free from disease." The coronary arteries were rather thicker than natural, and presented some white spots on their inner surface, but they were not ossified. "At the highest point of the arch of the aorta was an ossification, about the size of a shilling, shelving towards the edge, which, at some points, was sharp and irregular. Across this ran a band of cartilage, dividing the ossification into two portions, and forming a kind of hinge, by which these moved on each other. The ossification was exactly accommodated, in shape, to the vaulted form of the aorta. A small ossified portion was also found at the bifurcation of the vessel."

We have copied this note of the dissection from the MEDICAL GAZETTE, rather than trust to our memory, although we heard the paper read—because we know that our contemporary has access, like the COURIER, to exclusive information from high quarters. The note is perfectly correct, though somewhat abridged. The following passage, from the same source, will afford an idea of the pathological conclusions to which Dr. Warren came from this case.

"Dr. Warren is of opinion (as we understood) that when, in consequence of mental or bodily excitement, an additional quantity of blood was thrown into the aorta, so as to distend it, the shell of bone would, as it were, open on the cartilage as a hinge; but that, when the vessel contracted again, before it could return to its former curve, the sharp edge of the ossification would necessarily be pressed upon by its coats, which would thus be irritated—and hence, probably, the more violent paroxysms; while the situation of the morbid deposit, just at the point of the aorta most liable to be acted upon by the usual current of the blood, might account for the habitual aching."

When the paper was read, we certainly felt considerable disappointment—not only as to the anatomical details, but as to the pathological conclusions. Dr. Warren, as one of the heads of the profession, must take every opportunity of prosecuting morbid anatomy, and is therefore intimately acquainted with every deviation from natural structure. We are consequently bound to believe that, not only the heart, but the nerves leading to the heart, were in a state of perfect integrity, in this case. Still, our own limited experience in *post mortem* investigations would lead us to doubt that a scale of ossification, the size of a shilling, in the arch of the aorta, could have produced such dreadful paroxysms, and ultimately death, notwithstanding the hinge which was observed in this scale, and the "sharp edge" of the ossification, which was supposed to irritate the coats of the artery. Conceiving that the inner coats of arteries were supplied with nerves from the *ganglionic* system, we did not imagine that *pain* could have been thus produced, when the root of the aorta was inordinately filled with blood, "in consequence of mental or bodily excitement." In short, we cannot see any reasonable ground for attributing the dreadful paroxysms, with which this poor lady was affected, to the scale of ossification in the arch of the aorta. We look upon the case as a NEURALGIA of the heart and arteries, and conceive that the ossific deposit above-mentioned was a mere coincidence, and not a cause of the terrible symptoms which harrassed the patient for ten weeks, and then terminated her life.
